# Human pluripotent stem cell–derived neuronal progenitor cells promote neurogenesis and functional recovery by attenuating neuroinflammation and via extracellular vesicles in a subacute stroke model

**DOI:** 10.3389/fimmu.2025.1650092

**Published:** 2025-09-18

**Authors:** Hyun-Jin Kim, Dong Hee Kim, Jee In Choi, Hye Ryeong Sim, Sangyong Jung, Dong-Youn Hwang, MinYoung Kim

**Affiliations:** ^1^ Department of Biomedical Science, CHA University School of Medicine, Seongnam, Republic of Korea; ^2^ Rehabilitation and Regeneration Research Center, CHA University School of Medicine, Seongnam, Republic of Korea; ^3^ Department of Medical Science, College of Medicine, CHA University, Seongnam, Republic of Korea; ^4^ Department of Rehabilitation Medicine, CHA Bundang Medical Center, CHA University School of Medicine, Seongnam, Republic of Korea

**Keywords:** cell therapy, paracrine, neurorestoration, neurogenesis, neuroinflammation

## Abstract

**Introduction:**

Stroke remains a leading cause of long-term disability worldwide. The limited therapeutic window of current treatments underscores the need for alternative regenerative strategies. Neural progenitor cells (NPCs) are promising candidates for brain repair. However, the optimal timing of therapy and the mechanisms underlying its effects on ischemic stroke remain unclear.

**Methods:**

To evaluate their efficacy, NPCs were administered intravenously to a subacute stroke (day 7) rat model. The outcomes were determined by comparison with saline treatment using neurobehavioral assessments, infarct volume measurements, and molecular assays. In addition, NPC secretome profiling was performed to assess the underlying cellular mechanisms.

**Results:**

The NPC group showed significant improvement in motor function at 3 weeks post-injection (*p*<0.001) and reduced infarct volume (*p*<0.05). Immunofluorescence analysis revealed increased BrdU/DCX colocalization in the subventricular zone on day 14 (*p*<0.05). Western blotting confirmed the upregulation of NeuN (*p*<0.01), Nestin (*p*<0.05), and DCX (*p* = 0.0668) in the ipsilesional brain on day 28. The neuroprotection-related pathways revealed elevated protein levels of phosphorylated Akt, GSK3β, Erk, and CREB (*p*<0.05). Secretome profiling of NPC-conditioned medium and extracellular vesicles identified key regenerative factors such as Angiopoietin-1, BDNF, bFGF, MMP2, EGF, and VEGFa.

**Discussion:**

NPC administration during the subacute phase of stroke promoted functional recovery by promoting neurogenesis and modulating key survival pathways. These results highlight the therapeutic potential of NPCs as cell-based interventions for delayed stroke treatment and provide mechanistic insights into their reparative effects.

## 1 Introduction

Stroke affects millions of individuals annually, leading to significant neurological deficits and long-term disabilities (1). Despite extensive research efforts, thrombolytic therapy remains the only widely approved treatment, with a highly restricted therapeutic window (2). Consequently, patients with stroke have a critical unmet need for more effective and versatile treatment options. Among emerging strategies, cell therapy has received attention because of its potential to target multiple pathological mechanisms in stroke, with the ability to enhance intrinsic reparative processes in the brain (3). Various types of therapeutic cells have been explored to induce recovery from stroke. Mesenchymal stem cells and bone marrow-derived mononuclear cells have been studied extensively (3, 4). However, owing to their stronger affinity for neuronal lineage differentiation and greater capacity to support neural tissue regeneration, neural progenitor cells (NPCs) have been suggested to have the ability to induce neuronal recovery (5). This capacity contrasts with the major mechanisms of action of other cell types that exert immunomodulatory effects without direct effects on neural cells (6). Previous studies have demonstrated neural repair and enhanced neurogenic effects following the administration of NPC, which is also accompanied by neurobehavioral improvement in preclinical stroke models (7, 8).

Following a stroke, multiple endogenous recovery mechanisms are activated within the brain. Among these, neurogenesis, a process involving the differentiation of NPCs into mature neurons, is considered a promising approach for restoring damaged neural circuits (9). Although only a small fraction of progenitor cells can successfully differentiate into mature neurons and integrate into existing neuronal circuits, newly generated neurons may contribute to functional recovery (10, 11). The creation of a neurotrophic factor-enriched microenvironment has been reported to facilitate the recovery process, and NPCs have emerged as therapeutic candidates capable of establishing this supportive niche (12). Several sources of NPC may exert different efficacy according to the characteristics of each cell. In this study, we developed a pluripotent stem cell-derived NPC following a specific differentiation protocol that will have strong potency of neural regeneration (13). Therefore, this study aimed to evaluate both the therapeutic potential and the underlying mechanism of action. To understand their possible impact on therapeutic cells, the ingredients of the secretome should be verified. Previously, the efficacy of cell therapy was predicted under the assumption that transplanted cells would be engrafted and would directly replace damaged neurons (14). However, subsequent studies have demonstrated that most transplanted cells fail to survive long term and rarely persist beyond several days post-transplantation (10). Instead, the secretome, which is a collection of the principal bioactive molecules released by therapeutic cells, has been suggested as the principal mediator of therapeutic effects (15).

The target condition of this therapeutic research was stroke in the subacute phase according to clinical needs. Most studies have focused on the acute phase of therapeutic cell administration. However, during the acute phase, patients are frequently under unstable medical conditions to receive cell therapeutics, and predicting the outcome of neuronal impairment is difficult (16). This timing was selected not only in response to clinical needs but also to optimize the therapeutic potential of NPC transplantation. The subacute phase of stroke is characterized by the remaining potential for heightened plasticity in a receptive tissue environment, which may enhance the efficacy of cell-based therapies. This contrasts with the chronic phase, where reduced tissue repair plasticity limits the potential for functional recovery (7, 17). Therefore, the subacute stage was considered a temporally favorable window for the neuroregenerative efficacy of NPC therapy.

Accordingly, this study aimed to (1) evaluate the therapeutic efficacy of NPC treatment in a rodent stroke model during the subacute phase, (2) investigate the neurogenesis-promoting effects of NPC transplantation and elucidate the underlying molecular mechanisms, and (3) assess the secretome of NPC that might be associated with neuronal recovery.

## 2 Materials and methods

### 2.1 Animals

Male Sprague–Dawley rats (5–6 weeks, 160–180 g) were purchased from Samtako Bio Korea (Osan, South Korea). The animals were housed in groups of one or two per cage in a temperature-controlled environment (22 ± 2 °C), under a 12-hour light/dark cycle, with free access to food and water. The room humidity was maintained at 50% ± 10%. All animal experiments were performed following the guidelines of the CHA University Institutional Animal Care and Use Committee (approval no. 200104) and the CHA University standards for the ethical treatment of laboratory animals. The animals were allowed a 7-day acclimation period before initiating the experimental protocols. At the experimental endpoint, animals were euthanized using CO_2_ inhalation at a flow rate of 20% of the chamber volume per minute for 3 minutes, in accordance with institutional ethical guidelines. Cessation of breathing was visually confirmed, after which CO_2_ was administered for an additional ~1 minute to ensure death. Animal experiments were conducted for either 14 or 28 days following MCAO induction.

### 2.2 Middle cerebral artery occlusion modeling

Transient MCAO was induced using a 4–0 silicon-coated filament via intraluminal vascular occlusion. Rats were initially anesthetized with 3%–4% isoflurane, which was maintained at 2%–3% throughout the procedure. A midline cervical incision was made to expose the common carotid artery, which was then occluded using a 4–0 black silk suture. The external carotid artery (ECA) and internal carotid artery were then isolated, and a 30 mm-long 4–0 nylon suture filament with a 2–3 mm silicone-coated tip (0.39-mm diameter; Doccol, Sharon, MA, USA) was introduced into the ECA via micro-incision. The filament was advanced until its tip occluded the middle cerebral artery (MCA), and the ECA was ligated with silk sutures to prevent bleeding. The incision was closed, and the body temperature was maintained at 37 °C using a heating blanket during the 90-minute occlusion period. Subsequently, the surgical site was reopened, and the filament was carefully removed from the MCA to allow reperfusion. In the control group, rats underwent the same procedure as for MCAO modeling, including incision and CCA exposure, except for the silicon insertion process. The rats in the control group were not subjected to surgical intervention.

MCAO induction was confirmed by assessing neurological function using the modified Neurological Severity Score (mNSS) on post occlusion day 7. Only rats that scored between 8 and 12 (a total impairment score of 14) were included in the study. The animals were divided into three groups: a normal control group (control group), an MCAO group receiving sterile saline (saline group), and an MCAO group receiving NPCs (NPC group). Groups were randomly assigned and stratified based on body weight and mNSS scores to ensure balanced baseline characteristics. To evaluate the newly generated cells following NPC treatment, BrdU was administered at a dose of 100 mg/kg per day for 5 consecutive days, starting 3 days before NPC injection. MCAO induction was confirmed by assessing neurological function using the modified Neurological Severity Score (mNSS) on post occlusion day 7. Only rats that scored between 8 and 12 (a total impairment score of 14) were included in the study. The animals were divided into three groups: a normal control group (control group), an MCAO group receiving sterile saline (saline group), and an MCAO group receiving NPCs (NPC group). Groups were randomly assigned and stratified based on body weight and mNSS scores to ensure balanced baseline characteristics. To evaluate the newly generated cells following NPC treatment, BrdU was administered at a dose of 100 mg/kg per day for 5 consecutive days, starting 3 days before NPC injection.

### 2.3 Preparation of embryoid bodies derived from NPCs and treatment

Human embryonic stem cells (hESCs), derived from the H9 line (WiCell Research Institute, Madison, WI, USA), were dissociated using 2 mg/mL collagenase type IV (Worthington Biochemical Corporation, Lakewood, NJ, USA) at 37°C for 30 minutes. The detached hESCs were subsequently aggregated to form EBs and cultured in suspension for 4 days in Dulbecco’s modified eagle medium (DMEM)/F12 medium (Thermo Fisher Scientific, Waltham, MA, USA) supplemented with 10 µg/mL human insulin, 9 µg/mL transferrin, 14 ng/mL selenite, 5 µM PKCβ inhibitor, and 1 µM DMH1 (all from Sigma-Aldrich, St. Louis, MO, USA). The culture medium was replaced daily with a fresh medium. On day 4, EBs were transferred to Matrigel-coated culture dishes and maintained in NPC differentiation medium containing 1% N2 supplement (Thermo Fisher Scientific), 20 ng/mL basic fibroblast growth factor (bFGF, CHA Biotech, Pangyo, South Korea), and 25 µg/mL human insulin (Sigma-Aldrich). The medium was refreshed daily for the next 5 days to promote the formation of neural rosettes.

NPCs at passages 3 to 5 were immediately detached from the culture dishes prior to use, and 3 × 10^6^ cells were resuspended in 1 mL sterile saline. The cell suspension was administered was intravenously injected day 7 after MCAO induction. The control group received an equivalent volume of saline without cells.

NPCs were labeled with CM-DiI (Thermo Fisher Scientific), a red fluorescent lipophilic dye that integrates into the cell membrane. Following detachment from the culture dish, the cells were incubated with CM-DiI at a concentration of 1 µg per 1×10^6^ cells in 1 mL of suspension. The labeled cells were transplanted on day 7 post-MCAO.

### 2.4 Neurobehavioral assessment

Neurobehavior was assessed to evaluate neurological deficits in rats subjected to MCAO on days 1, 3, 7, 14, 21, and 28 post ischemia. All evaluations were performed by an investigator blinded to the group allocation. The primary assessment tool was the mNSS, a composite scoring system that quantifies sensory, motor, and reflex impairments, ranging from 0 to 14, with higher scores indicating greater neurological dysfunction (18). To ensure consistency in scoring, the research team established inter-rater reliability and achieved intraclass correlation coefficients exceeding 0.90 (19).

Additionally, the cylinder test was utilized to assess asymmetry in forelimb use between the contralateral and ipsilateral sides of the hemispheric lesion and was performed on the same days as the mNSS assessment. In this test, rats were placed in a transparent cylinder (20 cm in diameter and 30 cm in height) and their initial paw touches on the cylinder wall during rearing were recorded separately for each forelimb (contralateral, ipsilateral, or simultaneous bilateral touches) until 20 touches were observed. The cylinder test scores were calculated as follows:


$$\frac{I+(B/2)}{C}\;\times 100\left(\%\right)$$

where I, B, and C represent the number of touches made to the ipsilateral limb (I), contralateral limb (C), and both limbs (B), respectively. All behavioral tests were video-recorded by a designated investigator, and the recordings were later analyzed for scoring.

### 2.5 Sacrifice and tissue preparation

Rats were anesthetized with 2%–3% isoflurane and transcardially perfused with more than 100 mL of phosphate-buffered saline (PBS) for sacrifice. To prepare for the tissue examination, brains were carefully extracted from the skull and fixed in 4% paraformaldehyde at 4°C overnight. After a brief rinse with PBS, the brains were incubated in a 30% sucrose solution for 48 h for cryoprotection. Coronal sections were obtained using a cryostat with a thickness of 20 µm for immunofluorescence staining and 50 µm for cresyl violet staining.

### 2.6 Cresyl violet staining

Brain cryosections (50 µm thick) were air-dried to remove residual moisture. The sections were then washed in PBS for 10 min before being incubated in 0.1% cresyl violet solution for 15 min. Subsequently, the sections were dehydrated using a graded ethanol series (70%, 80%, 90%, 95%, and 100%), with each step lasting 1 min, followed by immersion in xylene for 5 min. After mounting with a mounting medium (Thermo Fisher Scientific), the slides were left to dry at room temperature overnight. Images were acquired using a Cytation 5 imaging system (Agilent Technologies, Santa Clara, CA, USA). The peri-infarct cortex was examined at higher magnification to identify condensed chromatin, which indicates nuclear damage. The infarct volume was quantified using the ImageJ software.

### 2.7 Immunofluorescence staining

Sections designated for immunofluorescence staining were washed in PBS for 10 min to remove residual optimal cutting temperature compounds. After removing excess PBS from the sections, a blocking solution containing 2% normal goat serum and 1% Triton X-100 in PBS was applied for 1 h at room temperature. Subsequently, the sections were incubated overnight at 4°C with primary antibodies, including NeuN (1:1000; Millipore, Bedford, MA, USA), BrdU (1:500; Novus, Centennial, CO, USA), Iba-1 (1:100; Abcam, Cambridge, UK), CD68 (1:200; Novus, Centennial, CO, USA), GFAP (1:200; Abcam, Cambridge, UK), and a mixture of Ki-67 (1:500; Abcam, Cambridge, UK) and Sox2 (1:500; Santa Cruz Biotechnology, Dallas, TX, USA), all diluted in 2% normal goat serum (NGS) blocking solution. Following primary antibody incubation, the sections were washed three times with PBS for 10 min each. Then, appropriate secondary antibodies (rabbit-488, mouse-488, rabbit-594, and mouse-594; all from Thermo Fisher Scientific, diluted 1:1000 in 2% NGS blocking solution) were applied for 1 h at room temperature. After secondary antibody incubation, the sections were washed three times with PBS and mounted using 4′,6-diamidine-2′-phenylindole dihydrochloride-containing mounting medium (Vector Laboratories, Burlingame, CA, USA) before being cover-slipped. Fluorescence images were acquired using a Nikon Eclipse Ts2 microscope (Nikon Instech, Tokyo, Japan).

### 2.8 Western blot analysis

After sacrificing 28 days post-MCAO induction, brains were carefully extracted. Then, ipsilesional hemispheres were separately homogenized in PBS on ice and subsequently lysed in T-PER lysis buffer (Thermo Fisher Scientific) supplemented with protease inhibitor (Sigma-Aldrich) and phosphatase inhibitor (Sigma-Aldrich).

Protein samples (20 μg) were loaded onto a sodium dodecyl-sulfate polyacrylamide gel electrophoresis gel and transferred onto a polyvinylidene fluoride membrane (Millipore, Bedford, MA, USA). The membrane was blocked with either 5% skim milk or 5% BSA solution for 1 h. Primary antibodies, including p-Akt (1:1000; Cell Signaling Technology, Danvers, MA, USA), Akt (1:1000; Cell Signaling Technology), p-Erk (1:1000; Cell Signaling Technology), Erk (1:1000; Cell Signaling Technology), p-GSK3β (1:1000; Cell Signaling Technology), GSK3β (1:1000; Cell Signaling Technology), p-CREB (1:1000; Millipore, Bedford, MA, USA), and β-actin (1:2000; Santa Cruz Biotechnology, Dallas, TX, USA), were incubated overnight at 4°C on a shaker.

Following incubation, the membrane was washed three times with Tris-buffered saline containing 0.1% Tween 20 detergent (TBST) buffer and then incubated with an horseradish peroxidase (HRP)-conjugated secondary antibody (rabbit and mouse HRP-antibody, Cell Signaling Technology) for 1 h at room temperature. After additional washes with TBST, protein bands were detected using an ECL solution (Millipore) and visualized using a Chemi-Doc imaging system (Atto Korea, Daejeon, South Korea).

### 2.9 Preparation of extracellular vesicles

EVs were purified from NPC-conditioned medium (CM) using a size-exclusion ultrafiltration technique involving Amicon^®^ Ultra-15 centrifugal filter units with a 100 kDa molecular weight cut-off (Millipore). This approach enabled the enrichment of vesicles larger than ~30 nm without ultracentrifugation. Initially, the CM was sequentially centrifuged at 300 × g for 10 min to eliminate cells, at 2,000 × g for 10 min to remove apoptotic bodies, and at 10,000 × g for 30 min at 4°C to clear large vesicles and cell debris. The resulting supernatant was passed through a 0.22 μm syringe filter (Millipore) to exclude particles exceeding 220 nm in diameter, including large microvesicles. The filtered medium was then loaded into Amicon Ultra-15 centrifugal units (100 kDa cut-off) and centrifuged at 4,000 × g for 20–30 min at 4°C, or until the retentate volume was reduced to approximately 200–500 μL. To enhance the purity and eliminate residual contaminants, the retained EV-rich fraction was washed with sterile PBS and centrifuged again under identical conditions. The final concentrated EV preparation was collected from the upper reservoir and either stored at −80°C for long-term preservation or used directly for downstream applications.

### 2.10 Secretome assay

The secretome of CM and EV derived from NPC was quantified using a 17-plex Luminex assay (Angiopoietin-1, BDNF, bFGF, MMP2, NRP2, EGF, VEGFa, TNFα, IL-1β, IGF, TPI, Tie2, Ang2, GDF14, IL-10, IGFBP, and TSP-2) on the Luminex 200 system (R&D Systems, USA). CM was collected from NPCs cultured for 48 h in the absence of EGF, bFGF, and N2 supplements to minimize their potential influence on the secretome profile. Following collection, the supernatant was filtered through a 0.2 μm membrane filter prior to analysis. EVs were isolated as described in Section 1.9. Standard curves were generated using the mean fluorescence intensity values obtained from the Luminex 200 system and fitted to a five-parameter logistic model to determine analyte concentrations. Each biological sample was measured in duplicate (technical replicates).

### 2.11 Statistical analysis

Statistical analyses were performed using Prism 8.0 (GraphPad Software Inc., CA, USA). Data are expressed as mean ± standard deviation. For comparisons between two groups, statistical significance was assessed using Student’s t-test. Normality was assessed using the Shapiro–Wilk test. Based on the normality results, parametric tests were applied to normally distributed data, whereas non-parametric Mann–Whitney U tests were used for data that did not meet the assumption of normality. Statistical significance was set at *p*<0.05.

## 3 Results

### 3.1 Intravenous administration of NPC improves functional recovery in stroke model

**Figure 1:**
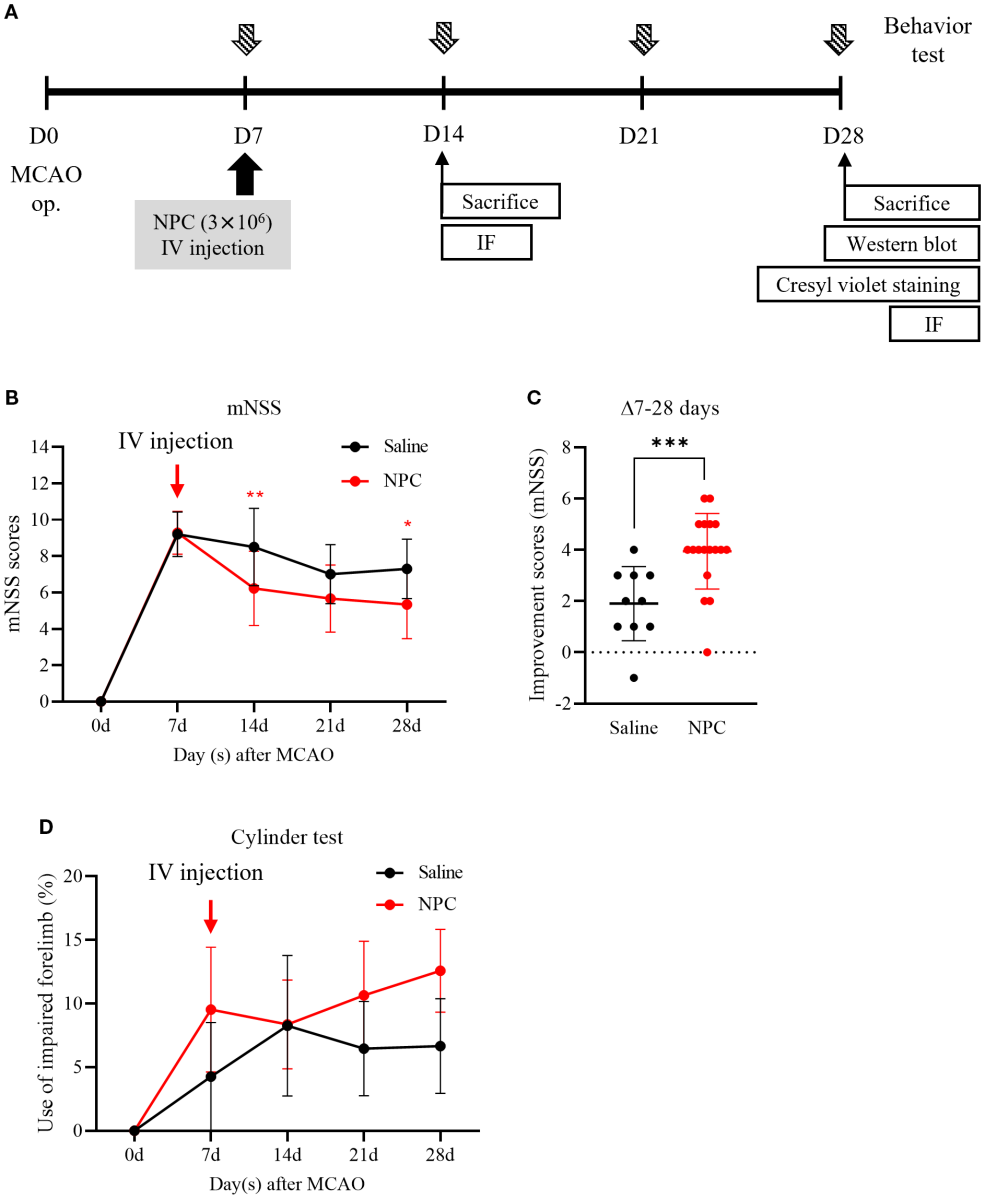
Behavior test after administration of NPC in middle cerebral artery occlusion (MCAO) rats. **(A)** Experimental scheme. **(B)** The mNSS test and **(D)** the cylinder test were conducted in saline (n = 10) and NPC (n = 18) groups. **(C)** The improvement score (mNSS) graph represents the reduction in mNSS scores between days 7 and 28 in each group. NPC, neural progenitor cell; mNSS, modified Neurological Severity Score. Data are presented as mean ± SD (n = 10–18 per group). Statistical analyses were performed as follows: in **(B)**, parametric tests (unpaired t-test) were used for days 14 and 21, and non-parametric tests (Mann–Whitney U test) for days 7 and 28; in **(C)**, all time points were analyzed using non-parametric tests; in **(D)**, non-parametric tests were applied at all time points. (*p < 0.05, **p < 0.01, ***p < 0.001 vs. saline.).

To target the subacute phase of stroke, NPCs were administered 7 days after MCAO induction. At this point, neurological impairment scores were used as reference values to ensure that the saline and NPC groups were assigned with comparable levels of neurological deficits. To enhance their clinical applicability, NPCs were delivered via intravenous injection. The effect of NPC administration on functional recovery was assessed using the mNSS to evaluate overall neurobehavioral function and the cylinder test to assess upper limb motor function at 7-day intervals up to 28 days post-injection (**Figure 1A**). The NPC-treated group demonstrated a significant reduction in mNSS scores compared to the saline-treated group on day 14 (saline: 8.50 ± 2.12, NPC: 6.22 ± 2.05, *p*<0.01) and on day 28 (saline: 7.30 ± 1.64, NPC: 5.33 ± 1.88, *p*<0.05) (**Figure 1B**). Additionally, the change in mNSS scores from days 7 to 28 demonstrated a significant difference between the two groups (saline: 1.90 ± 1.37, NPC: 3.94 ± 1.43, *p*<0.001), suggesting that NPC administration meaningfully improved functional recovery in the MCAO model (**Figure 1C**). Although the usage rate of the impaired forelimb, as assessed by the cylinder test, slightly increased over 3 weeks post-NPC administration, no significant improvement was observed compared with the saline-treated group (**Figure 1D**).

### 3.2 NPC administration reduces infarct volume

**Figure 2:**
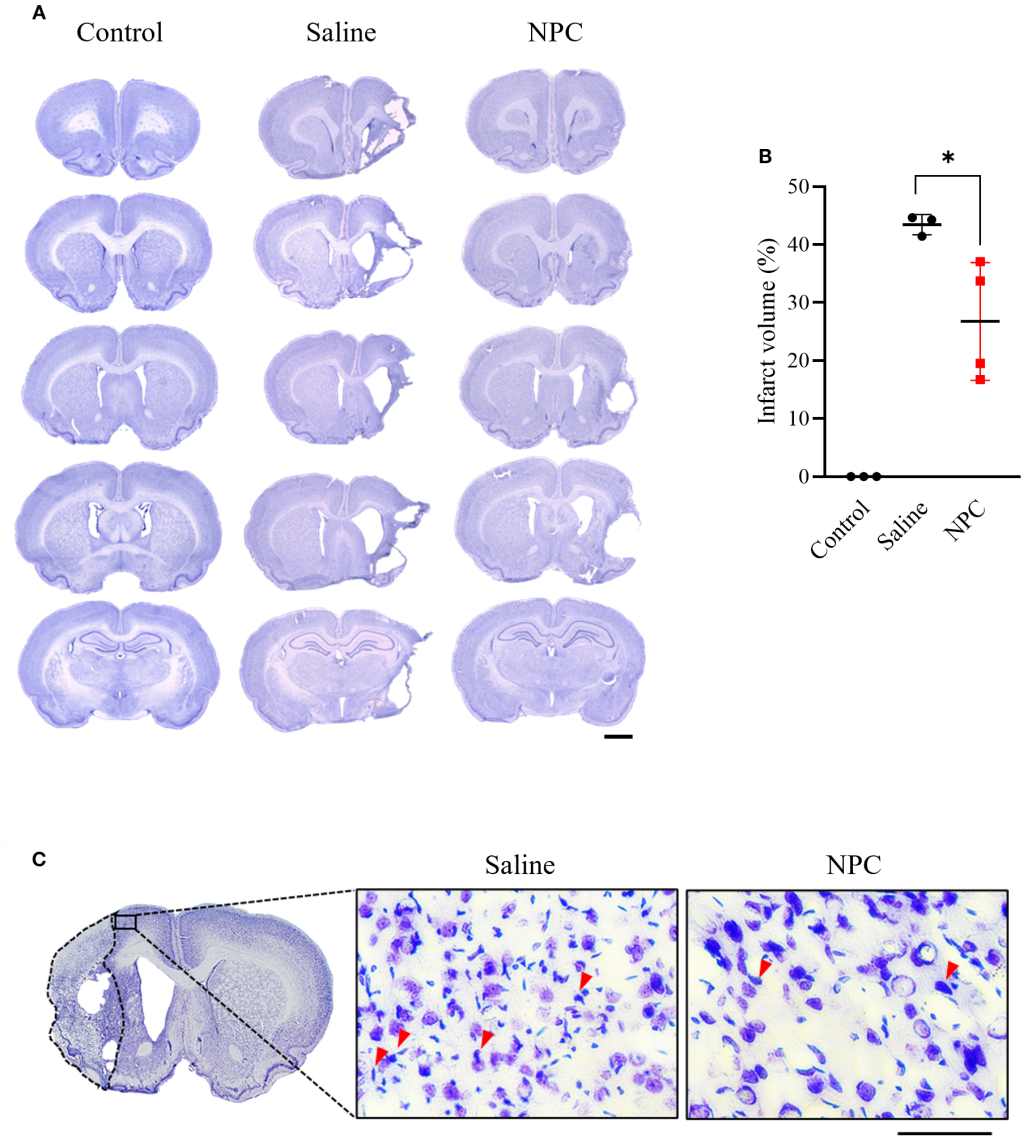
Infarct volume was assessed by cresyl violet staining of coronal brain section slice at day 28 after MCAO. **(A)** Representative stained brain section of each group in the saline and NPC groups. Scale bar = 2000μm. **(B)** Quantitative analysis of cresyl violet staining to measure infarct volume for the saline group (n=3) and NPC group (n=4). **(C)** A magnified image of the peri-infarct region in cresyl violet-stained brain tissue, showing neurons and chromatin condensation. NPC, neural progenitor cell; MCAO, middle cerebral artery occlusion. Scale bar = 50μm. (n = 3~4 rats per group, unpaired t-test; vs. Saline, *p < 0.05, **p < 0.01),.

To evaluate the effect of NPC administration on brain damage, infarct volume was quantified using cresyl violet staining. On day 28 post-MCAO, infarct volume of the ipsilesional hemisphere was significantly reduced in the NPC-treated group compared to the saline-treated group (Saline: 43.44 ± 1.76, NPC: 26.76 ± 10.13, *p*<0.05) (**Figure 2B**). Furthermore, examination of the peri-infarct region revealed a reduction in chromatin condensation, a hallmark of neuronal apoptosis, in the NPC-treated group compared to the saline-treated group. Overall, these findings suggest that NPC administration mitigated brain damage after MCAO.

### 3.3 NPC administration enhances neurogenesis in the subventricular zone

**Figure 3:**
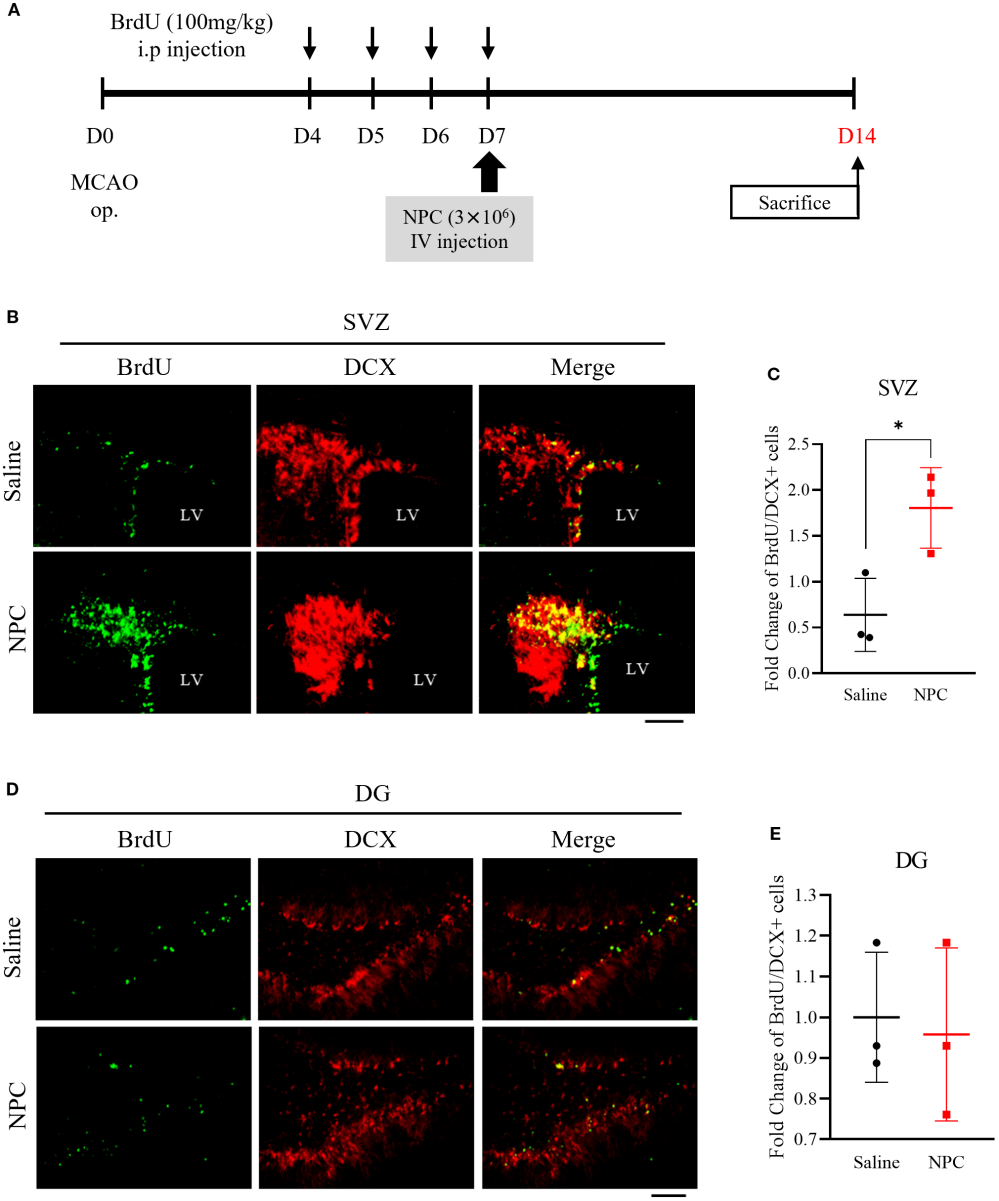
Assessment of neurogenesis via BrdU staining following NPC administration in the MCAO brain. **(A)** Schematic representation of the BrdU injection protocol in MCAO models. **(B)** Immunofluorescence images showing BrdU and DCX co-staining in the subventricular zone (SVZ). Scale bar = 100μm. **(C)** Quantification of BrdU+/DCX+ cells in the SVZ of the saline-treated (n = 3) and NPC-treated groups (n = 3). **(D)** Immunofluorescence images showing BrdU and DCX co-staining in the dentate gyrus (DG). Scale bar = 100μm. **(E)** Quantification of BrdU+/DCX+ cells in the DG of the saline-treated (n = 3) and NPC-treated groups (n = 3). NPC, neural progenitor cell; MCAO, middle cerebral artery occlusion. Data are presented as mean ± SD (n = 3–4 rats per group, unpaired t-test; *p < 0.05 vs. Saline).

To investigate the effect of NPC administration on neurogenesis, BrdU was administered, and the BrdU and DCX expressions in the SVZ and dentate gyrus (DG) were analyzed by immunofluorescence staining. BrdU was intraperitoneally injected at a dose of 100 mg/kg once daily for 4 consecutive days prior to NPC administration. On day 7 post-NPC administration, the number of proliferating cells labeled with BrdU and co-localized with DCX, an immature neuronal marker, was significantly increased in the SVZ of the NPC-treated group compared to that in saline-treated group (**Figures 3B, C**, saline: 1 ± 0.69, NPC: 1.80 ± 0.36, *p*<0.05). However, in the DG, no significant difference in BrdU/DCX co-localization was observed between the saline and NPC-treated groups (**Figures 3D, E**, saline: 1 ± 0.13, NPC: 0.96 ± 0.17). This lack of response in the DG may be attributed to its lower sensitivity to stroke-induced and NPC-mediated neurogenesis than the SVZ. Thus, although NPC administration did not significantly alter neurogenesis in the DG, it enhanced neurogenesis in the SVZ.

### 3.4 NPC administration increases neuronal population and upregulates neurogenesis-related markers in the peri-infarct region

**Figure 4:**
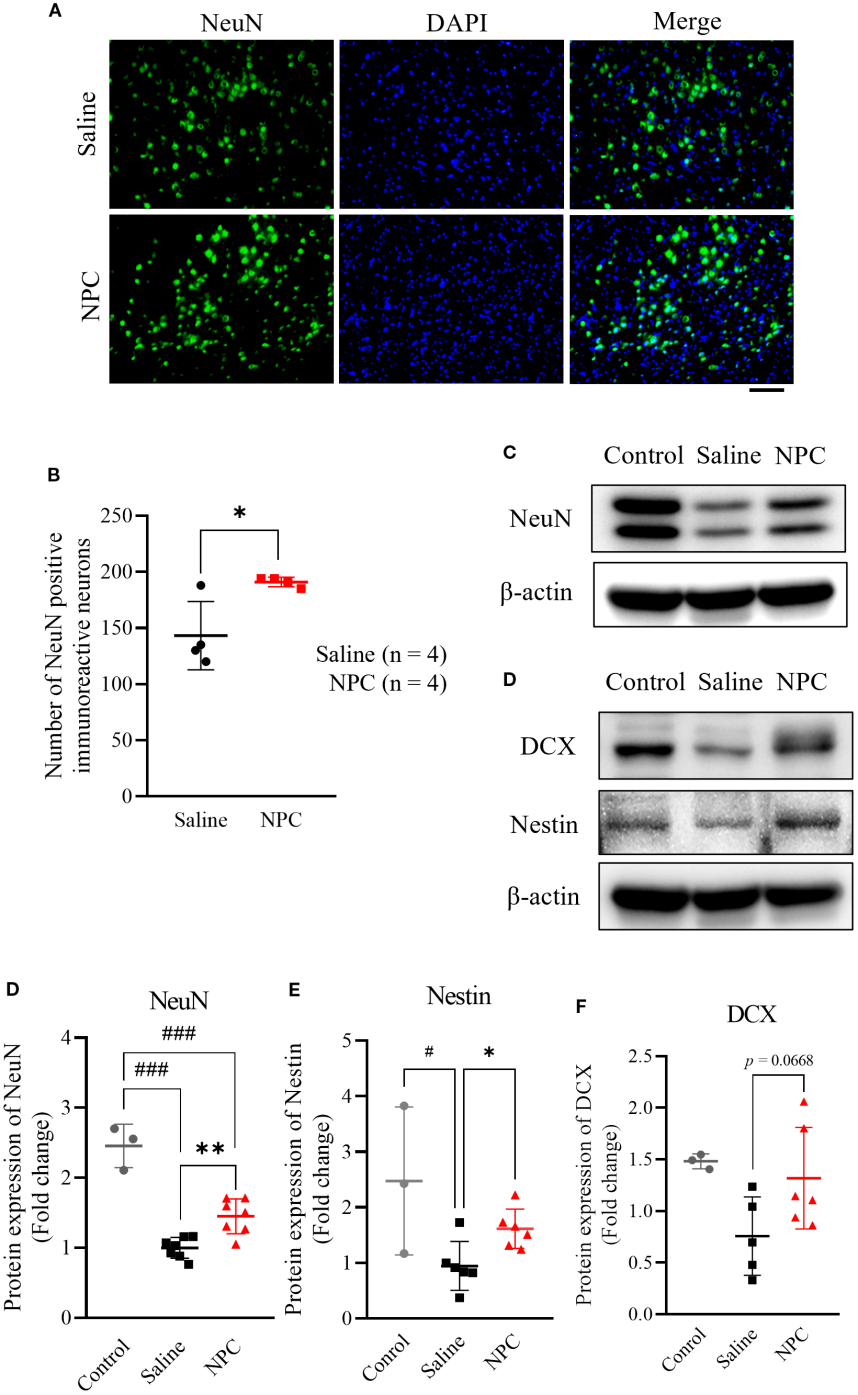
Evaluation of neurogenesis after NPC administration. **(A)** ImmunoFluorescence staining of NeuN in the peri-infarct on 28 day after inducing MCAO. **(B)** Quantification of NeuN positive cells in the peri-infarct region of the saline (n=4) and NPC groups (n=4). Scale bar = 100μm. **(C)** Western blot data of neurogenesis markers including NeuN **(D)**, nestin **(E)**, and DCX **(F)**. NPC, neural progenitor cell; MCAO, middle cerebral artery occlusion. Data were presented as mean ± SD (n = 3~4 rats per group, unpaired t-test; vs. Saline, *p < 0.05, **p < 0.01; vs. Control, #*p* < 0.05, ###*p* < 0.001.

To verify whether the BrdU-based findings were reflected in NeuN expression, we performed immunofluorescence staining for NeuN in the peri-infarct cortex to assess its expression pattern. On day 28, the expression of the mature neuronal marker NeuN was examined in the peri-infarct area. The NPC-treated group exhibited a 1.33-fold increase in NeuN-positive signals (**Figures 4A, B**; *p*<0.05) compared with the saline-treated group, which was statistically significant and consistent with the Western blot results (**Figures 4C, D**; *p*<0.05). Additionally, the neurogenesis markers nestin (**Figures 4C, E**; *p*<0.05) and DCX (**Figures 4C, F**; *p*<0.001) were significantly upregulated by 1.71-fold and 2.83-fold, respectively, compared with that in the saline-treated group. Thus, NPC administration stimulated neurogenesis and contributed to the increase in the number of neurons observed on day 28.

### 3.5 NPC effects achieved by mediation of neurogenesis-associated signaling pathways

**Figure 5:**
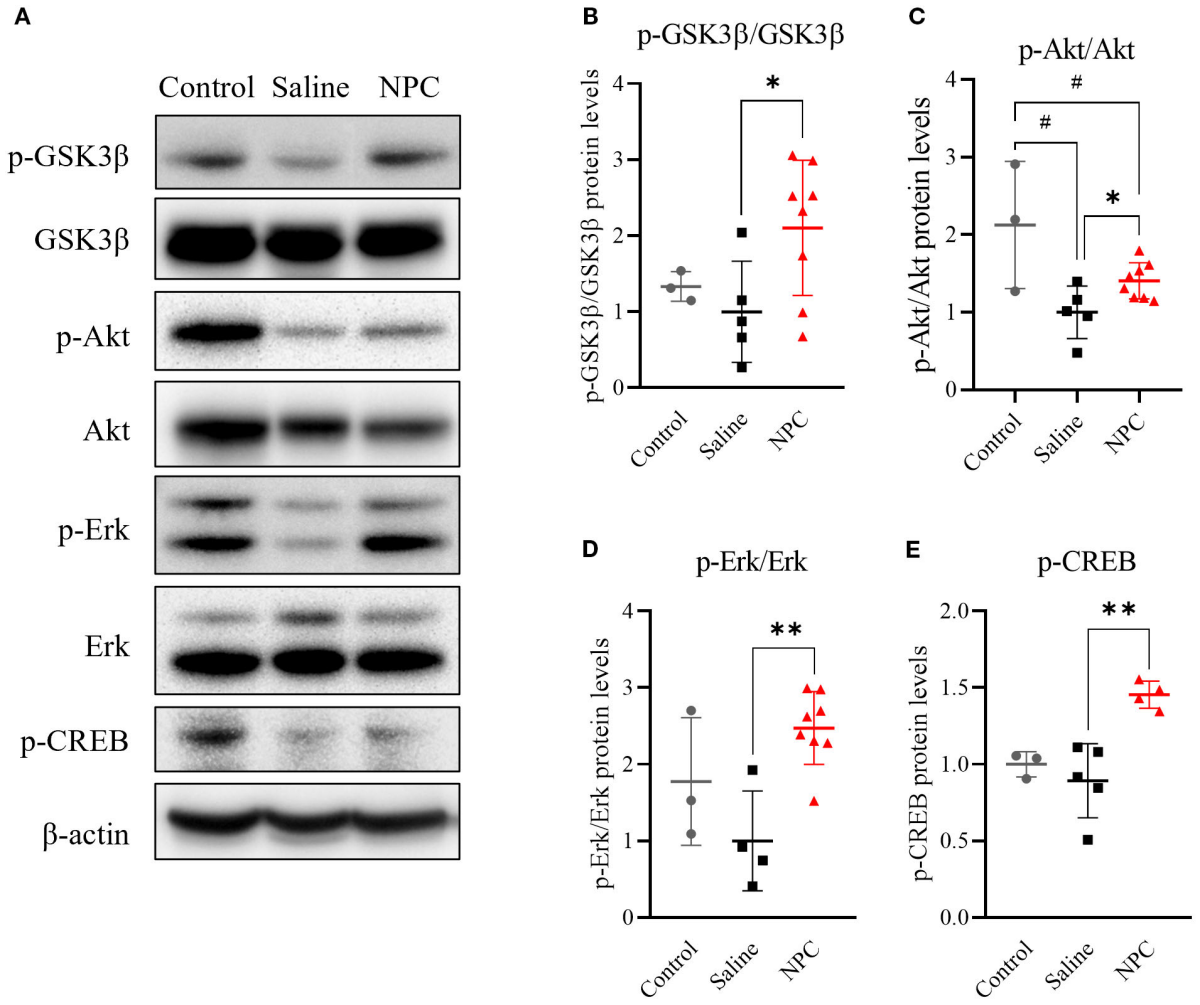
Western blot analysis of neurogenesis-related signaling pathways in ipsilateral brain samples at day 28. **(A, D, F)** Western blot results showing phosphorylated GSK3β (p-GSK3β), Akt (p-Akt), Erk (p-Erk), and CREB (p-CREB) in ipsilateral brain samples collected on day 28 post-MCAO. **(B, C, E, G)** Quantification of western blot data for p-GSK3β **(B)**, p-Akt **(C)**, p-Erk **(E)**, and p-CREB **(G)**. MCAO, middle cerebral artery occlusion. Data are presented as mean ± SD (n = 3–4 rats per group, unpaired t-test; *p < 0.05, **p < 0.01 vs. Saline, #*p* < 0.05, vs. Control).

Following NPC injection, behavioral performance improved, neurogenesis increased, and microglial activation was attenuated. To investigate the underlying mechanisms, various signaling pathways associated with neurogenesis, including Akt, ERK, GSK-3β, and BDNF, were analyzed in the D28 brain using western blotting. An increase in phosphorylated Akt (**Figures 5A, C**; *p*<0.05) was observed in the NPC group, accompanied by elevated levels of phosphorylated GSK3β (**Figures 5A, B**; *p*<0.05) and potentially phosphorylated CREB (**Figures 5F, G**; *p*<0.01). Additionally, phosphorylated ERK signals were increased (**Figures 5D, E**; *p*<0.01), which contributed to CREB phosphorylation independently of Akt. By contrast, no significant changes were observed in BDNF, MEK, or mTOR levels (data not shown). Thus, the therapeutic efficacy of NPCs may be mediated through the activation of neurogenesis-related pathways, specifically via enhanced phosphorylation of Akt and ERK. This in turn leads to CREB phosphorylation, translocation to the nucleus, and induction of neurogenesis-associated gene transcription, thereby contributing to the observed therapeutic effects.

### 3.6 NPC administration attenuates microglial activation in the peri-infarct region

**Figure 6:**
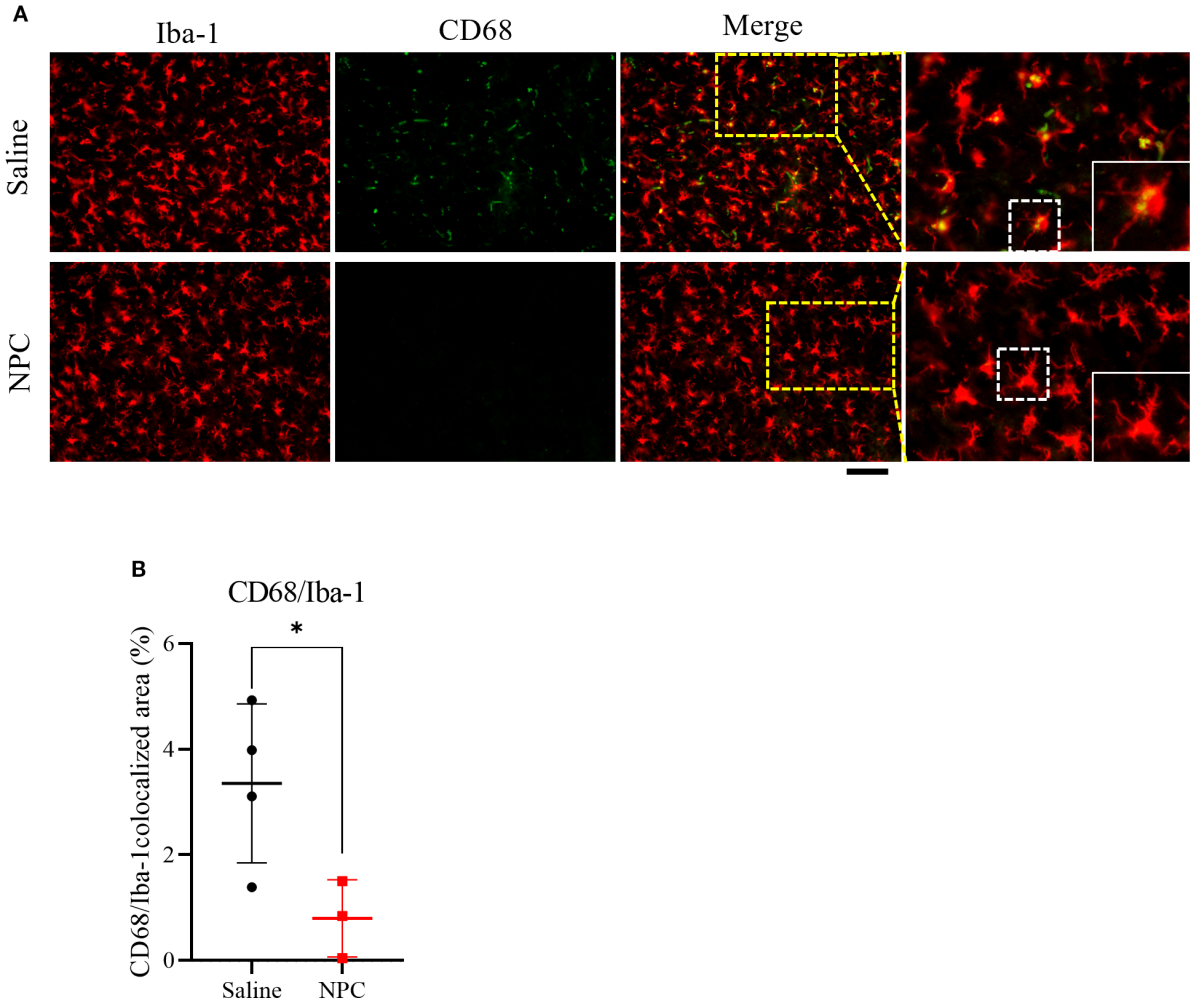
Assessment of microglial activation following NPC treatment. **(A)** Representative immunofluorescence images showing co-localization of Iba-1 and CD68 in the peri-infarct region at 28 days after MCAO. Scale bar = 100μm. **(B)** Quantification of the CD68^+^ area within the Iba-1^+^ microglial population. Data are presented as mean ± SD (n = 3–4 rats per group, unpaired t-test (n = 3–4 rats per group, unpaired t-test, *p < 0.05).

Based on previous studies demonstrating that the alleviation of inflammation can positively influence neurogenesis (20), we hypothesized that the anti-inflammatory effect of NPCs may represent an additional mechanism by which they enhance neurogenesis. To investigate whether NPC administration could attenuate stroke-induced inflammation, we examined microglial activation in the brain at 28 days post-stroke (**Figures 6A, B**). Immunofluorescence staining for Iba-1, a general microglial marker, and CD68, a marker of activated microglia, revealed that the co-localized area of CD68 within Iba-1^+^ microglia was significantly reduced in the NPC-treated group (0.80 ± 0.73%) compared to the saline group (3.26 ± 1.67%). Moreover, Iba-1^+^ microglia in the NPC group exhibited a more ramified morphology, indicative of a less activated state. These findings suggest that NPC treatment may help modulate the post-stroke inflammatory environment in the brain.

### 3.7 NPC releases neurogenesis-related secretome

**Figure 7:**
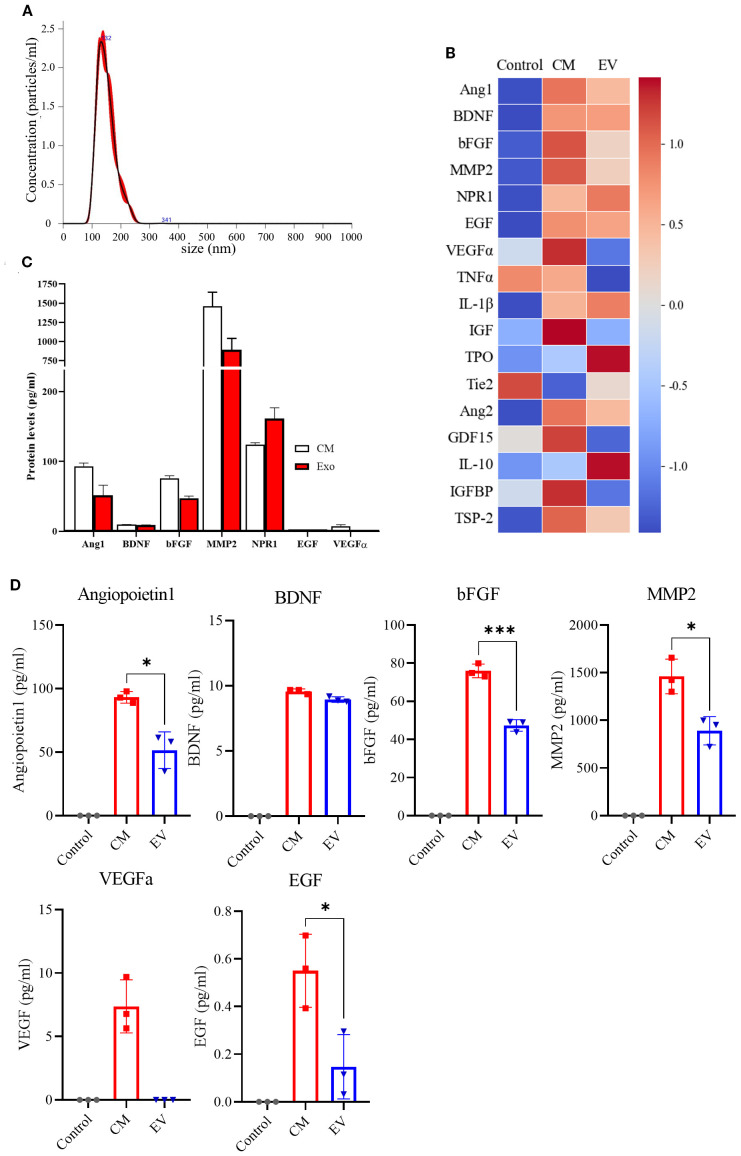
Secretome Assay of NPC-Conditioned Media. **(A)** Representative of EVs, showing the size distribution and particle concentration. **(B)** Heat map displaying the secretome assay results for 17 neurogenesis-related markers in the control group (DMEM/F-12, n = 3), NPC-conditioned media (CM, n = 3), and extracellular vesicles (EVs) isolated from CM (n = 3). **(C)** Quantification graphs of secretome assay markers across experimental groups. **(D)** Individual quantification graphs for each marker assessed in the secretome assay. (n = 3 per group, Mann–Whitney U test; vs. Saline, *p < 0.05, ***p < 0.001),.

To identify neurogenesis-related proteins secreted by NPCs, a secretome assay was conducted using 17 candidate proteins known to be associated with neurogenesis as follows: Angiopoietin-1, BDNF, bFGF, MMP2, NRP2, EGF, VEGFa, TNFα, IL-1β, IGF, TPI, Tie2, Ang2, GDF14, IL-10, IGFBP, and TSP-2. Previous studies have demonstrated the therapeutic effects of EV administration in stroke models. To determine the extent to which EVs contribute to the NPC-derived secretome, we separated and analyzed the NPC-CM and EV-enriched fractions derived from the CM (EV). Among the 17 candidate proteins, seven (Angiopoietin-1, BDNF, bFGF, MMP2, Neuropilin-1, EGF, and VEGFa) were significantly upregulated in the NPC-CM compared to the control medium (DMEM/F-12) (**Figures 7A, B**), suggesting a potential role of the NPC-derived secretome in promoting neurogenesis. Additionally, analysis of the total secretome revealed that >50% of several key proteins were present in the EV fraction (**Figures 7B, C**), including Ang1 (55.29%), BDNF (93.45%), bFGF (62.28%), MMP2 (61.02%), and EGF (26.75%). By contrast, VEGFa was not detected in the EV fraction. Thus, more than half of the neurotrophic factors secreted by NPCs were released via EVs, suggesting that the proportion of each protein transported through EV may vary.

## 4 Discussion

Stroke has inherent treatment limitations, including a narrow therapeutic time window and an insufficient capacity for neural regeneration. Various cell-based therapies, including NPC therapies, have been actively investigated to address the issues of time sensitivity and limited neuroregenerative potential. NPCs, which are more lineage-committed for neural differentiation than other stem cell types, have shown promising effects on neuronal recovery. Previous research that administered NPCs via the intracerebroventricular route has reported the formation of synaptic connections with host striatal neurons and a reduction in infarct volume (21, 22). However, these studies on NPC administration for therapeutic purposes are difficult to translate into clinical use. First, most of these methods rely on invasive delivery methods, such as direct intracerebral injection (23). Second, the studies employed culture conditions involving animal serum (24). To overcome these limitations, we adopted an intravenous administration route that is more feasible for clinical use and utilized xeno-free cultured NPCs to facilitate translational relevance and potential clinical applications. The NPCs used in this study were differentiated using a novel protocol developed by Hwang and cultured under xeno-free conditions. The differentiation method was designed to enhance the neurogenic potential and ensure safety. Although the dual SMAD inhibition method that use transforming growth factor beta (TGF-β) and bone morphogenetic protein inhibitors has been widely adopted in reported NPC differentiation protocols (25), the NPCs used in our study were generated using a Protein Kinase C beta (PKC-β) inhibitor instead of a TGF-β inhibitor (13). Inhibition of the TGF-β pathway results in undesirable downstream effects including negative effect on cell survival (26). PKC-β suppresses neurogenesis (27) and is associated with gliogenesis (28). Accordingly, inhibition of PKC-β can promote neural lineage commitment while suppressing glial differentiation, thereby creating a more favorable environment for maintaining neuronal fate. This approach offers the advantages of achieving higher neural differentiation efficiency and generating a more homogeneous population of NPCs.

The timing of treatment is a critical component of the therapeutic strategy and should be carefully determined based on the evolving pathological environment following stroke onset. In this context, the subacute phase represents a strategically advantageous window for cell-based therapies following stroke. The subacute phase follows the acute phase and is characterized by intense inflammatory responses and reactive oxygen species-mediated damage (29). During this phase, various endogenous repair mechanisms, including neurogenesis, are activated and the brain exhibits relatively high levels of neural plasticity (30). During the acute phase, the administration of therapeutic cells may be adversely affected by a hostile microenvironment, including excessive inflammation and tissue damage (31). Therefore, drug administration during the subacute phase may enhance cell survival and therapeutic efficacy. Nevertheless, most stroke therapies developed to date have primarily focused on the acute phase, whereas research targeting the subacute phase remains relatively limited. Given its clinical demand and translational potential, the subacute phase has therapeutic significance, although it remains insufficiently explored. Based on these considerations, we selected the subacute phase as the time point for NPC administration.

In brief, we examined the effect of intravenously administered NPC, which were differentiated following a specific protocol, on neuronal recovery in a subacute stroke model reflecting clinically relevant features, including a therapeutically appropriate time window and delivery route. First, stroke animals treated with NPCs showed improvement in neurobehavior with a significantly greater reduction in mNSS score compared to those treated with saline. Importantly, behavioral recovery, which emerged from day 14, was maintained through day 28 post-stroke, which was the final time point of assessment. Previous studies have shown that behavioral improvements following NPC transplantation in the subacute phase can be maintained for up to 84 days post-stroke, suggesting that the therapeutic benefits observed at day 28 in our study are likely to persist beyond this time point (7). However, unlike the mNSS, the cylinder test did not reveal significant differences between groups. This discrepancy could be attributed to the distinct sensitivities of the tests; the mNSS evaluates gross neurological deficits across multiple domains, whereas the cylinder test is more specific to asymmetric forelimb use (32). Gross motor recovery often precedes fine motor improvements (33), suggesting that, although NPC therapy may effectively restore general neurological function, more specialized interventions or extended recovery periods might be necessary to achieve fine motor recovery (34).

NPC administration not only led to significant improvements in neurobehavior but also reduced the volume of infarcted brain tissue. Furthermore, decreased chromatin condensation observed in neurons around the infarct area indicates that NPCs are likely to exert neuroprotective effects by attenuating neuronal damage. During the subacute phase, the administered NPCs are presumed to secrete various neurotrophic factors that suppress the progression of injury initiated during the acute phase and facilitate the stable integration of newly generated neurons in the peri-infarct region. The results of the present study also indicate a possible NPC-induced enhancement of neurogenesis in the brain. NPC administration significantly increased the proliferation of BrdU-labeled neural precursor cells in the SVZ, indicating enhanced neurogenesis. By contrast, no such effect was observed in the DG, suggesting regional differences in the neurogenic response to NPC treatment. The SVZ is anatomically closer to the ischemic lesion and responds more sensitively to external stimuli, such as growth factors and inflammation (35). The anatomical proximity of the SVZ and its heightened sensitivity could explain the stronger neurogenic effects observed in the SVZ following NPC transplantation. Conversely, the DG, which lacks a direct connection to the injury site, constitutes a more stable neurogenic niche and tends not to exhibit rapid neurogenic activation after stroke (36). Thus, NPCs selectively enhanced endogenous regenerative processes in the SVZ, rather than in the DG. Moreover, the increased numbers of NeuN-positive cells and elevated nestin and DCX expression levels in the peri-infarct region further support the role of NPC transplantation in promoting neurogenesis and neuronal survival.

**Figure 8:**
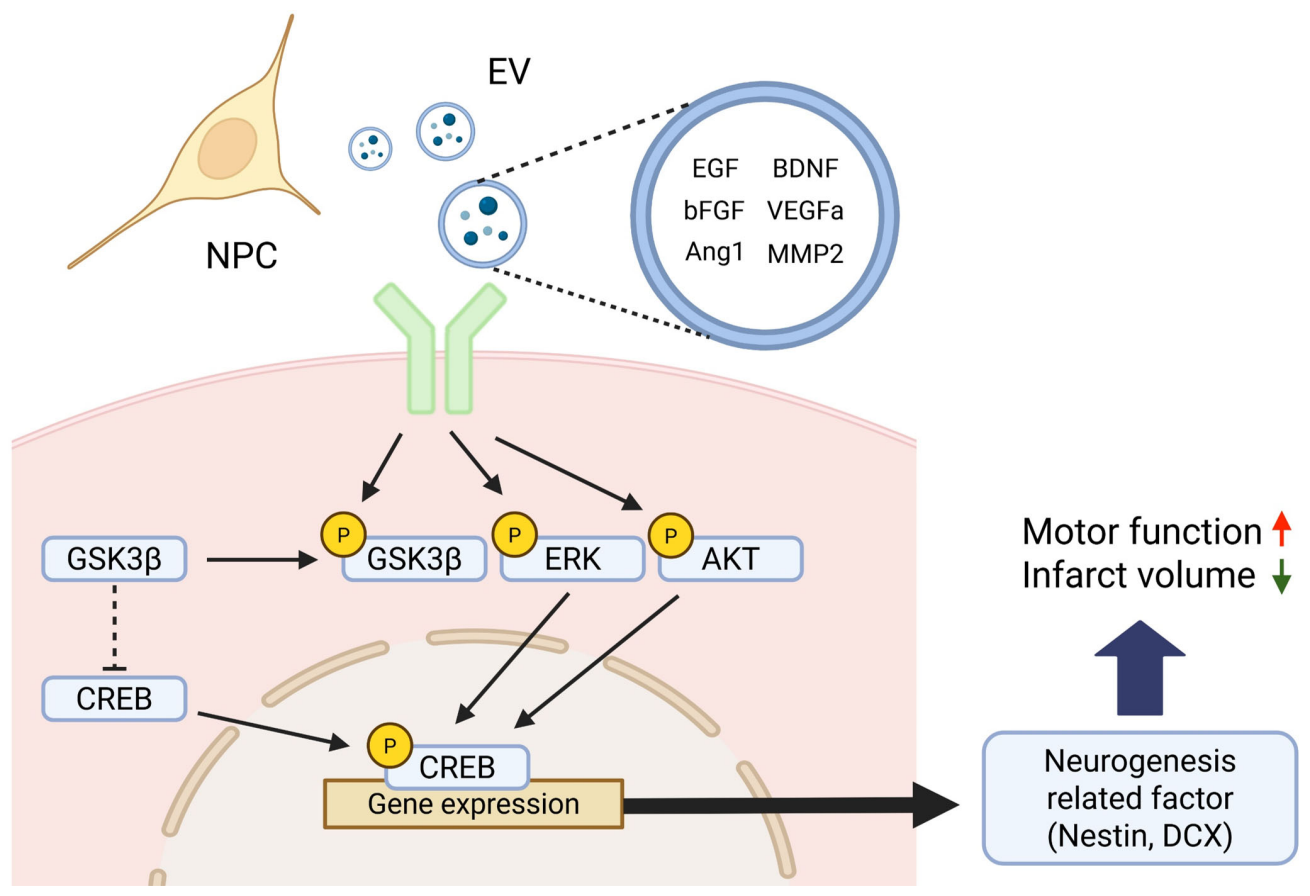
Schematic illustration of the proposed signaling mechanism underlying NPC-induced neurogenesis after stroke. Neural progenitor cells (NPCs) secrete various neurotrophic and angiogenic factors—including EGF, bFGF, Angiopoietin-1 (Ang1), BDNF, VEGFa, and MMP2—either as free soluble molecules or encapsulated within extracellular vesicles (EVs). These factors activate intracellular signaling cascades such as the Akt and Erk pathways upon binding to their respective receptors. Activated Akt phosphorylates and inhibits GSK3β, thereby relieving its repressive effect on CREB. Concurrently, Erk and Akt signaling promote CREB phosphorylation, which in turn facilitates the transcription of neurogenesis-related genes such as *Nestin* and *DCX*. This coordinated activation of CREB-dependent gene expression contributes to enhanced neurogenesis and functional recovery observed after NPC transplantation in the subacute phase of stroke. Created in BioRender. Kim, H. (2025).

At the molecular level, NPC transplantation is associated with an increased phosphorylation of Akt and ERK, two well-established signaling pathways regulate neurogenesis and neuronal survival (37). Moreover, elevated phosphorylation of CREB, a downstream transcription factor of both the AKT and ERK pathways, suggests the activation of gene programs involved in neuronal differentiation, synaptic plasticity, and cell survival (38, 39). In this context, GSK3β, which normally acts to inhibit CREB phosphorylation, becomes inactivated upon its own phosphorylation, thereby facilitating CREB activation. Thus, the concurrent promotion of CREB phosphorylation and inhibition of CREB-suppressive signaling likely act synergistically to enhance the expression of neurogenesis-related genes (40). This convergence of signaling pathways ultimately upregulates the expression of key neurogenic markers, including nestin and DCX, as confirmed by immunofluorescence staining and western blot analysis (**Figure 8**). Importantly, no significant changes were detected in endogenous BDNF expression in brain tissue, implying that the observed functional improvements were more likely attributable to NPC-derived paracrine effects rather than intrinsic BDNF upregulation. To further substantiate the involvement of these signaling pathways, future studies may incorporate pharmacological inhibition of Akt and ERK signaling. Such experiments could help determine whether activation of these pathways is functionally required for NPC-mediated neurogenesis and behavioral recovery.

Neuroinflammation has been shown to play a dual role in the regulation of neurogenesis following cerebral ischemia. While acute inflammation may initially act as a trigger for neurogenic activation, persistent or unresolved inflammation is known to exert deleterious effects, ultimately suppressing neurogenesis and impairing brain repair. Accordingly, targeting inflammation has emerged as a promising therapeutic strategy to enhance endogenous neurogenesis in the injured brain. NPCs have been reported to modulate the inflammatory response by interacting with resident immune cells such as microglia. Consistent with previous studies, our results demonstrated a reduction in the co-localized area of Iba-1 and CD68, which are markers of activated microglia, in the peri-infarct region of NPC-treated animals. This attenuation of microglial activation suggests that NPC administration mitigates chronic inflammation within the brain microenvironment. Given that chronic inflammation suppresses the proliferation and differentiation of neural precursor cells, this anti-inflammatory effect may contribute to the increased neurogenesis observed in the subventricular zone (SVZ) following NPC treatment. Therefore, our findings support the notion that NPCs enhance neurogenesis through both direct mechanisms—such as neurotrophic factor secretion—and indirect mechanisms mediated by the resolution of neuroinflammation. These results underscore the therapeutic potential of NPCs in restoring the regenerative capacity of the post-stroke brain by modulating the inflammatory environment.

Although previous studies primarily employed direct intracerebral delivery of NPCs, we adopted an intravenous route to enhance clinical translatability. Despite concerns regarding the limited ability of intravenously delivered cells to cross the blood–brain barrier (BBB) which was also observed in this study in the absence of NPCs in the ischemic brain region (**Supplementary Figure 1A**), our results demonstrate that systemically delivered NPCs could exert substantial effects on the brain microenvironment and significantly improve neurobehavioral outcomes. Overall, NPCs may exert their therapeutic effects not through direct engraftment at the lesion site, but rather via the secretion of neurotrophic factors capable of crossing or modulating the BBB. Our secretome analysis revealed that NPCs secrete a broad array of neurogenic and angiogenic factors, including Angiopoietin-1, BDNF, bFGF, MMP2, Neuropilin-1, and VEGFa (41, 42). These factors are known to activate receptor tyrosine kinases and subsequently stimulate downstream intracellular cascades, such as the Akt and ERK pathways (43), which is consistent with our molecular findings. However, most of these factors have limited ability to cross the BBB, posing a challenge to their direct delivery into the brain parenchyma.

A substantial portion of neurotrophic and angiogenic factors, including BDNF, bFGF, and VEGFα, was encapsulated within EVs, despite EVs representing only a subset of the CM. EVs protect their molecular cargo from enzymatic degradation and cross the BBB via endocytosis by endothelial cells, thereby facilitating targeted delivery into the brain (44). This suggests that EVs may serve as effective carriers, facilitating targeted delivery of therapeutic factors into the brain even after systemic administration (45, 46). Proteomic profiling revealed that many therapeutic factors were enriched in the EV fraction, and NPC-derived EVs are particularly associated with pathways related to EV biogenesis and vesicle-mediated transport (47), supporting the idea that EVs play a pivotal role in mediating the neuroprotective and regenerative effects of NPCs through selective enrichment and efficient delivery of bioactive molecules.

However, this study has some limitations. First, although we demonstrated increased neurogenesis and neuronal survival, we did not directly assess whether the newly generated neurons were functionally integrated into preexisting neural circuits. This remains an important question, as functional integration is considered essential for sustained behavioral recovery. Future studies may explore this aspect using approaches such as lineage tracing and electrophysiological assessments to evaluate synaptic incorporation and activity of NPC-derived neurons. Second, although we identified key therapeutic proteins encapsulated within the EVs, we could not directly confirm their ability to cross the BBB and reach the brain parenchyma. Follow-up studies employing labeled EVs or protein-specific tracking methods will be needed to address this. Lastly, this study utilized a single NPC source and animal model, which limited the generalizability of our findings. Additional validation using NPCs derived from different origins as well as larger and more biologically diverse animal models accounting for variables, such as sex and age and comorbidities including conditions like diabetes and hypertension, will be necessary to better assess the translational potential of this approach.

In conclusion, NPC transplantation during the subacute phase of stroke promoted functional recovery, reduced infarct volume, and enhanced neurogenesis through the activation of neurogenesis-related signaling pathways and paracrine mechanisms. In addition, our findings suggest that NPC-mediated attenuation of chronic inflammation may further contribute to the enhancement of neurogenesis by creating a more favorable microenvironment for endogenous repair. These findings highlight the therapeutic potential of NPCs in stroke treatment. Moreover, modulation of the secretome and EV-mediated delivery may serve as promising strategies for future clinical translation.

## Data Availability

The datasets presented in this study can be found in online repositories. The names of the repository/repositories and accession number(s) can be found in the article/**Supplementary Material.**

## References

[1] Feigin VL Brainin M Norrving B Martins SO Pandian J Lindsay P. World stroke organization: global stroke fact sheet 2025. Int J Stroke. (2025) 20:132–44. doi: 10.1177/17474930241308142, PMID: 39635884 PMC11786524

[2] Gunkan A Ferreira MY Vilardo M Scarcia L Bocanegra-Becerra JE Cardoso LJC. Thrombolysis for ischemic stroke beyond the 4.5-hour window: A meta-analysis of randomized clinical trials. Stroke. (2025) 56:580–90. doi: 10.1161/STROKEAHA.124.048536, PMID: 39882605

[3] Zhou L Zhu H Bai X Huang J Chen Y Wen J. Potential mechanisms and therapeutic targets of mesenchymal stem cell transplantation for ischemic stroke. Stem Cell Res Ther. (2022) 13:195. doi: 10.1186/s13287-022-02876-2, PMID: 35551643 PMC9096773

[4] Bang OY Kim EH Cha JM Moon GJ. Adult stem cell therapy for stroke: challenges and progress. J Stroke. (2016) 18:256–66. doi: 10.5853/jos.2016.01263, PMID: 27733032 PMC5066440

[5] Bacigaluppi M Pluchino S Peruzzotti-Jametti L Kilic E Kilic U Salani G. Delayed post-ischaemic neuroprotection following systemic neural stem cell transplantation involves multiple mechanisms. Brain. (2009) 132:2239–51. doi: 10.1093/brain/awp174, PMID: 19617198

[6] Drago D Cossetti C Iraci N Gaude E Musco G Bachi A. The stem cell secretome and its role in brain repair. Biochimie. (2013) 95:2271–85. doi: 10.1016/j.biochi.2013.06.020, PMID: 23827856 PMC4061727

[7] Doeppner TR Kaltwasser B Teli MK Bretschneider E Bahr M Hermann DM. Effects of acute versus post-acute systemic delivery of neural progenitor cells on neurological recovery and brain remodeling after focal cerebral ischemia in mice. Cell Death Dis. (2014) 5:e1386. doi: 10.1038/cddis.2014.359, PMID: 25144721 PMC4454329

[8] Belayev L Hong SH Menghani H Marcell SJ Obenaus A Freitas RS. Docosanoids promote neurogenesis and angiogenesis, blood-brain barrier integrity, penumbra protection, and neurobehavioral recovery after experimental ischemic stroke. Mol Neurobiol. (2018) 55:7090–106. doi: 10.1007/s12035-018-1136-3, PMID: 29858774 PMC6054805

[9] Zhao LR Willing A. Enhancing endogenous capacity to repair a stroke-damaged brain: An evolving field for stroke research. Prog Neurobiol. (2018) 163-164:5–26. doi: 10.1016/j.pneurobio.2018.01.004, PMID: 29476785 PMC6075953

[10] Christie KJ Turnley AM. Regulation of endogenous neural stem/progenitor cells for neural repair-factors that promote neurogenesis and gliogenesis in the normal and damaged brain. Front Cell Neurosci. (2012) 6:70. doi: 10.3389/fncel.2012.00070, PMID: 23346046 PMC3548228

[11] Shu H Guo Z Chen X Qi S Xiong X Xia S. Intracerebral transplantation of neural stem cells restores manganese-induced cognitive deficits in mice. Aging Dis. (2021) 12:371–85. doi: 10.14336/AD.2020.0717, PMID: 33815871 PMC7990353

[12] Hur HJ Lee JY Kim DH Cho MS Lee S Kim HS. Conditioned medium of human pluripotent stem cell-derived neural precursor cells exerts neurorestorative effects against ischemic stroke model. Int J Mol Sci. (2022) 23:17. doi: 10.3390/ijms23147787, PMID: 35887140 PMC9319001

[13] Park M Kim HM Shin HA Lee SH Hwang DY Lew H. Human pluripotent stem cell-derived neural progenitor cells promote retinal ganglion cell survival and axon recovery in an optic nerve compression animal model. Int J Mol Sci. (2021) 22:8. doi: 10.3390/ijms222212529, PMID: 34830410 PMC8622638

[14] Janowski M Wagner DC Boltze J. Stem cell-based tissue replacement after stroke: factual necessity or notorious fiction? Stroke. (2015) 46:2354–63. doi: 10.1161/STROKEAHA.114.007803, PMID: 26106118 PMC4519410

[15] Kawabori M Kuroda S Shichinohe H Kahata K Shiratori S Ikeda S. Intracerebral transplantation of MRI-trackable autologous bone marrow stromal cells for patients with subacute ischemic stroke. Med. (2024) 5:432–44 e4. doi: 10.1016/j.medj.2024.02.009, PMID: 38547868

[16] Savitz SI Misra V Kasam M Juneja H Cox CS Jr. Alderman S. Intravenous autologous bone marrow mononuclear cells for ischemic stroke. Ann Neurol. (2011) 70:59–69. doi: 10.1002/ana.22458, PMID: 21786299

[17] O’Shea TM Ao Y Wang S Wollenberg AL Kim JH Ramos Espinoza RA. Lesion environments direct transplanted neural progenitors towards a wound repair astroglial phenotype in mice. Nat Commun. (2022) 13:5702. doi: 10.1038/s41467-022-33382-x, PMID: 36171203 PMC9519954

[18] Schaar KL Brenneman MM Savitz SI. Functional assessments in the rodent stroke model. Exp Transl Stroke Med. (2010) 2:13. doi: 10.1186/2040-7378-2-13, PMID: 20642841 PMC2915950

[19] Yu J Moon J Jang J Choi JI Jung J Hwang S. Reliability of behavioral tests in the middle cerebral artery occlusion model of rat. Lab Anim. (2019) 53:478–90. doi: 10.1177/0023677218815210, PMID: 30482088

[20] Ekdahl CT Claasen JH Bonde S Kokaia Z Lindvall O. Inflammation is detrimental for neurogenesis in adult brain. P Natl Acad Sci USA. (2003) 100:13632–7. doi: 10.1073/pnas.2234031100, PMID: 14581618 PMC263865

[21] Noh JE Oh SH Lee S Lee S Kim YH Park HJ. Intracerebral transplantation of HLA-homozygous human iPSC-derived neural precursors ameliorates the behavioural and pathological deficits in a rodent model of ischaemic stroke. Cell Prolif. (2020) 53:e12884. doi: 10.1111/cpr.12884, PMID: 32713053 PMC7507302

[22] Jin K Xie L Mao X Greenberg MB Moore A Peng B. Effect of human neural precursor cell transplantation on endogenous neurogenesis after focal cerebral ischemia in the rat. Brain Res. (2011) 1374:56–62. doi: 10.1016/j.brainres.2010.12.037, PMID: 21167824 PMC3057169

[23] Hong S Lee SE Kang I Yang J Kim H Kim J. Induced neural stem cells from human patient-derived fibroblasts attenuate neurodegeneration in Niemann-Pick type C mice. J Vet Sci. (2021) 22:e7. doi: 10.4142/jvs.2021.22.e7, PMID: 33522159 PMC7850792

[24] Tekkatte C Gunasingh GP Cherian KM Sankaranarayanan K. Humanized” stem cell culture techniques: the animal serum controversy. Stem Cells Int. (2011) 2011:504723. doi: 10.4061/2011/504723, PMID: 21603148 PMC3096451

[25] Zhang M Ngo J Pirozzi F Sun YP Wynshaw-Boris A. Highly efficient methods to obtain homogeneous dorsal neural progenitor cells from human and mouse embryonic stem cells and induced pluripotent stem cells. Stem Cell Res Ther. (2018) 9:67. doi: 10.1186/s13287-018-0812-6, PMID: 29544541 PMC5856210

[26] Deng Z Fan T Xiao C Tian H Zheng Y Li C. TGF-beta signaling in health, disease, and therapeutics. Signal Transduct Target Ther. (2024) 9:61. doi: 10.1038/s41392-024-01764-w, PMID: 38514615 PMC10958066

[27] Geribaldi-Doldan N Gomez-Oliva R Dominguez-Garcia S Nunez-Abades P Castro C. Protein kinase C: targets to regenerate brain injuries? Front Cell Dev Biol. (2019) 7:39. doi: 10.1186/s13287-018-0812-6, PMID: 30949480 PMC6435489

[28] Garcia-Bernal F Geribaldi-Doldan N Dominguez-Garcia S Carrasco M Murillo-Carretero M Delgado-Ariza A. Protein kinase C inhibition mediates neuroblast enrichment in mechanical brain injuries. Front Cell Neurosci. (2018) 12:462. doi: 10.3389/fncel.2018.00462, PMID: 30542270 PMC6277931

[29] Candelario-Jalil E Dijkhuizen RM Magnus T. Neuroinflammation, stroke, blood-brain barrier dysfunction, and imaging modalities. Stroke. (2022) 53:1473–86. doi: 10.1161/STROKEAHA.122.036946, PMID: 35387495 PMC9038693

[30] Cocco C Siotto M Guerrini A Germanotta M Galluccio C Cipollini V. Systemic oxidative stress in subacute stroke patients undergoing rehabilitation treatment. Antioxidants (Basel). (2024) 13. doi: 10.3390/antiox13030354, PMID: 38539887 PMC10967715

[31] Boese AC Le QE Pham D Hamblin MH Lee JP. Neural stem cell therapy for subacute and chronic ischemic stroke. Stem Cell Res Ther. (2018) 9:154. doi: 10.1186/s13287-018-0913-2, PMID: 29895321 PMC5998588

[32] Shi X Bai H Wang J Wang J Huang L He M. Behavioral assessment of sensory, motor, emotion, and cognition in rodent models of intracerebral hemorrhage. Front Neurol. (2021) 12:667511. doi: 10.3389/fneur.2021.667511, PMID: 34220676 PMC8248664

[33] van Lieshout ECC Boonzaier J Pel AJ van Heijningen CL Vink JJ Visser-Meily JMA. Translational value of skilled reaching assessment in clinical and preclinical studies on motor recovery after stroke. Neurorehabil Neural Repair. (2021) 35:457–67. doi: 10.1177/15459683211005022, PMID: 33825580 PMC8127668

[34] Yamashita T Sasaki M Sasaki Y Nagahama H Oka S Kataoka-Sasaki Y. Rehabilitation facilitates functional improvement following intravenous infusion of mesenchymal stem cells in the chronic phase of cerebral ischemia in rats. Brain Res. (2024) 1825:148709. doi: 10.1016/j.brainres.2023.148709, PMID: 38072373

[35] Cuartero MI Garcia-Culebras A Torres-Lopez C Medina V Fraga E Vazquez-Reyes S. Post-stroke neurogenesis: friend or foe? Front Cell Dev Biol. (2021) 9:657846. doi: 10.3389/fcell.2021.657846, PMID: 33834025 PMC8021779

[36] Ceanga M Dahab M Witte OW Keiner S. Adult neurogenesis and stroke: A tale of two neurogenic niches. Front Neurosci. (2021) 15:700297. doi: 10.3389/fnins.2021.700297, PMID: 34447293 PMC8382802

[37] Qin C Yang S Chu YH Zhang H Pang XW Chen L. Signaling pathways involved in ischemic stroke: molecular mechanisms and therapeutic interventions. Signal Transduct Target Ther. (2022) 7:215. doi: 10.1038/s41392-022-01064-1, PMID: 35794095 PMC9259607

[38] Kitagawa K. CREB and cAMP response element-mediated gene expression in the ischemic brain. FEBS J. (2007) 274:3210–7. doi: 10.1111/j.1742-4658.2007.05890.x, PMID: 17565598

[39] Caracciolo L Marosi M Mazzitelli J Latifi S Sano Y Galvan L. CREB controls cortical circuit plasticity and functional recovery after stroke. Nat Commun. (2018) 9:2250. doi: 10.1038/s41467-018-04445-9, PMID: 29884780 PMC5993731

[40] Hoffmeister L Diekmann M Brand K Huber R. GSK3: A kinase balancing promotion and resolution of inflammation. Cells. (2020) 9:8. doi: 10.3390/cells9040820, PMID: 32231133 PMC7226814

[41] Petcu EB Smith RA Miroiu RI Opris MM. Angiogenesis in old-aged subjects after ischemic stroke: a cautionary note for investigators. J Angiogenes Res. (2010) 2:26. doi: 10.1186/2040-2384-2-26, PMID: 21110846 PMC3000373

[42] Liu Y Zhang H Yan L Du W Zhang M Chen H. MMP-2 and MMP-9 contribute to the angiogenic effect produced by hypoxia/15-HETE in pulmonary endothelial cells. J Mol Cell Cardiol. (2018) 121:36–50. doi: 10.1016/j.yjmcc.2018.06.006, PMID: 29913136

[43] Song YY Liang D Liu DK Lin L Zhang L Yang WQ. The role of the ERK signaling pathway in promoting angiogenesis for treating ischemic diseases. Front Cell Dev Biol. (2023) 11:1164166. doi: 10.3389/fcell.2023.1164166, PMID: 37427386 PMC10325625

[44] Li C Qin S Wen Y Zhao W Huang Y Liu J. Overcoming the blood-brain barrier: Exosomes as theranostic nanocarriers for precision neuroimaging. J Control Release. (2022) 349:902–16. doi: 10.1016/j.jconrel.2022.08.002, PMID: 35932883

[45] Wiklander OPB Brennan MA Lotvall J Breakefield XO El Andaloussi S. Advances in therapeutic applications of extracellular vesicles. Sci Transl Med. (2019) 11:3. doi: 10.1126/scitranslmed.aav8521, PMID: 31092696 PMC7104415

[46] Nieland L Mahjoum S Grandell E Breyne K Breakefield XO. Engineered EVs designed to target diseases of the CNS. J Control Release. (2023) 356:493–506. doi: 10.1016/j.jconrel.2023.03.009, PMID: 36907561 PMC10226535

[47] Campero-Romero AN Real FH Santana-Martinez RA Molina-Villa T Aranda C Rios-Castro E. Extracellular vesicles from neural progenitor cells promote functional recovery after stroke in mice with pharmacological inhibition of neurogenesis. Cell Death Discov. (2023) 9:272. doi: 10.1038/s41420-023-01561-4, PMID: 37507361 PMC10382527

