# Supplementary material for: Human pluripotent stem cell–derived neuronal progenitor cells promote neurogenesis and functional recovery by attenuating neuroinflammation and via extracellular vesicles in a subacute stroke model
Source: Front Immunol. 2025 Sep 18;16:1650092. doi: 10.3389/fimmu.2025.1650092 (PMC12488434; doi:10.3389/fimmu.2025.1650092)
Supplement: Supplementary file 1 [file Supplementaryfile1.docx]

Supplementary Material


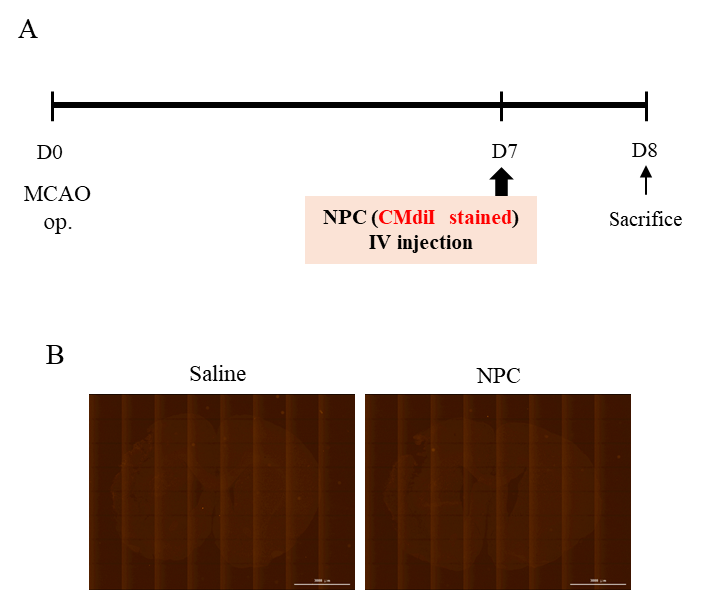


Supplementary Figure 1. Verification NPC in the brain after administration of NPC in middle cerebral artery occlusion (MCAO) rats (A) The experimental scheme. (B) Whole brain Montage of CM-diI stained NPC injection on 8 days afer MCAO inducing


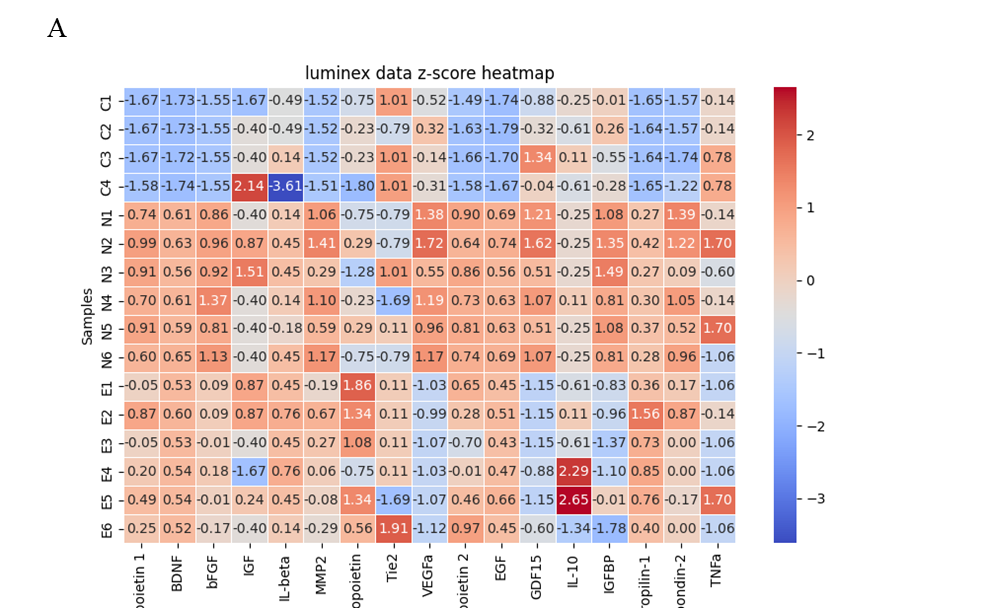


**Supplementary Figure 2.** Heat Map of Individual Data from Each Group in the Secretome Assay


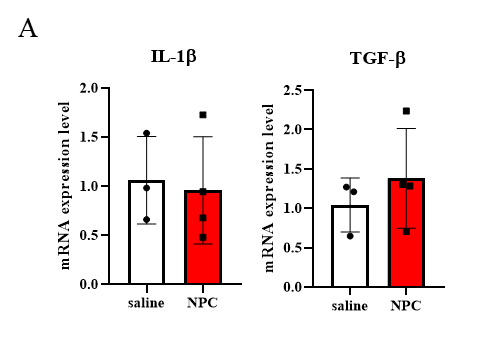


**Supplementary Figure 3** Inflammatory Response in MCAO Following NPC Administration (A) qPCR analysis of inflammatory markers IL-1β and TGF-β in the ipsilateral brain at day 10 post-MCAO.
